# Rabies in Alaska, from the past to an uncertain future

**DOI:** 10.1080/22423982.2018.1475185

**Published:** 2018-05-15

**Authors:** Karsten Hueffer, Molly Murphy

**Affiliations:** Department of Veterinary Medicine, University of Alaska Fairbanks, Fairbanks, Alaska, USA

**Keywords:** Rabies, Alaska

## Abstract

Rabies is a serious zoonotic disease with significant public health consequences in the circumpolar North. Recent studies have advanced our understanding of the disease ecology in Alaska. In this paper, we review historical records of rabies in Alaska ranging from the late nineteenth century to the present, analyse the public health impact in the state and review studies on disease ecology before assessing challenges and anticipated altered disease dynamics in the face of a rapidly changing North. Rabies is a disease that has been present in Alaska continuously for over 100 years. It is maintained in bats and foxes with the arctic fox likely playing a bigger role in maintaining the virus, although a multi-host system with both red and arctic foxes cannot be excluded. Some modelling evidence suggest a possible decrease in rabies due to a changing climate, although uncertainty is high around these predictions for rabies distribution in Alaska into the future.

## Introduction

The rabies virus is a member of the genus *Lyssavirus* in the family Rhabdoviridae, which causes a zoonotic disease that is nearly 100% fatal once symptoms occur. Most domestic and free-ranging mammalian species are susceptible to this neurotropic virus, which causes severe neurological manifestations in affected individuals, including (among other clinical signs) behavioural changes and paralysis followed by death typically within days. The virus is present in the saliva and neural tissue of affected animals and is typically transmitted via bite wound, or the entrance of infectious saliva into an existing break in the skin. It poses a significant public health challenge especially in underdeveloped regions of the world, due to under-vaccination of companion animals and a lack of access to post-exposure prophylactic treatment. Estimates vary on the total impact, owing to insufficient diagnosis and reporting infrastructure in the most affected regions. Dog-transmitted rabies causes the highest human burden; canine-transmitted rabies virus kills approximately 59,000 humans in developing countries and causes economic losses of more than $8 billion USD annually [1]. In developed countries, canine rabies is largely controlled through vaccination of dogs; however, wildlife-maintained rabies often remains a significant problem for public health authorities [2]. In response, rabies has been successfully controlled in wildlife through oral rabies vaccination campaigns in some regions [3]. The virus is maintained in various wildlife hosts worldwide. In North America, skunks (especially *Mephitis mephitis*), raccoon*s* (*Procyon lotor*) and foxes (*Vulpes vulpes* and *Vulpes lagopus*) are the main hosts of terrestrial rabies [2]. In most areas of the world, bats serve as maintenance hosts for many lyssaviruses, including rabies virus. Each host species maintains distinct variants of the rabies virus; however, the mechanism of adaptation of rabies virus variants to their respective hosts is currently not well understood.

## Rabies in the Arctic

In the Arctic, a rabies-like neurological disease has been described for over 100 years [4]. However, a lack of specific diagnostic methods and a weak reporting infrastructure make the interpretation of these disease reports difficult in that canine distemper morbillivirus can cause a disease in wild and domestic canids which has similar clinical signs. Most early descriptions of a rabies-like disease in dogs date from the first half of the twentieth century [5–7]. Some of these early studies mention the cyclical nature of disease outbreaks as well as an apparent seasonality, with most cases reported in the winter months. The earliest experimental studies in the 1940s confirmed rabies virus infection in foxes and dogs [6,7]. Rabies is distributed throughout the circumpolar North, with only the Nordic nations of Finland, Sweden and Norway considered to be rabies free [4]. The arctic fox (*V. lagopus*) and possibly the red fox (*V. vulpes*) are considered the main hosts within the arctic region [4]. Dogs currently do not play a significant role as maintenance hosts due to the decline of sled dog populations in rural areas, and the introduction of vaccinations against rabies which have reduced the number of susceptible dogs throughout the Arctic. However, the lack of veterinary care in remote areas still poses a challenge throughout the Arctic. Many dogs remain unvaccinated and can pose a significant threat by potentially exposing humans after contact with rabid wildlife.

A single rabies virus variant (Arctic rabies), which reacts with monoclonal antibody P-41 in antibody typing assays, has been identified throughout the Arctic. More recent studies using genetic sequencing tools have identified four variants within the Arctic rabies strain. Some variants have been further divided in subgroups [8]. Arctic rabies variant 1(Arctic-1) is found in southern Ontario as a result of spread from the northern part of that province and establishment in red foxes. Arctic rabies variant 2 (Arctic-2) is found in Eastern Russia and in Alaska on the Seward Peninsula. Arctic rabies variant 3 (Arctic-3) has a circumpolar distribution, while Arctic rabies variant 4 (Arctic-4) is found only in southwest Alaska [9].

Rabies viruses in the Arctic pose a threat to more populated regions as well, as demonstrated by the spread of Arctic-1 into southern Ontario [10]. In that outbreak (involving skunks, raccoons and foxes), reports of disease stretched from the outbreak origin in the high Canadian Arctic to the US–Canadian border region in the second half of the twentieth century. Significant resources were required to control this outbreak via the oral vaccination of wildlife. Challenging sampling logistics and the high cost of testing hinder progress in understanding the biology of rabies in the sparsely populated regions of the planet. While oral vaccines licensed for use in wildlife are safe and effective in arctic foxes [11–14], large-scale vaccination in this species is not cost effective and has not been achieved.

## Historical perspective

Historical reports on a rabies-like disease in the Arctic are sporadic and are based largely on reports of “mad dogs” or “mad foxes”. Nelson provided the oldest known description of rabies in Alaska in 1887 [15]. In his report, Nelson describes a disease in sled dogs that is similar to “madness of dogs in lower latitudes….”. Interestingly, Nelson does not describe this disease in other canids. Adolph Murie reports on an outbreak of rabies in red foxes in the lower Kuskokwim country in 1907, when Foxes were very abundant [16]. A 1915 outbreak of rabies in foxes was also described in the Yukon Delta [17].

Historical reports of rabies in humans in Alaska are rare. In 1914, a human case was described in an adult male following a bite from a sled dog in Candle in northwest Alaska [17]. The next cases, as reported by Rausch [18] date from 1942 to 1943, occurred following an attack by a wolf, and the presence of rabies in the dogs of the communities of Noorvik and Wainwright. In spite of frequent reports of rabies in foxes and sled dogs, the incidence of rabies in humans in Alaska is quite low. Several hypotheses have been formed to explain this discrepancy (especially given the close and frequent contact between humans, sled dogs and foxes). First, as most cases occur during the winter months, the heavy clothing worn in the North during this season might offer protection from animal bites. Alternatively, it has been speculated that the circulating virus strain may be less infective for humans [18], although data in support of this speculation are currently lacking. For example, one study described a high anti-rabies antibody titre in an unvaccinated trapper in Alaska who trapped foxes [19]. Presumably repeated exposure induced an immune response without resulting in clinical disease.

The first report in the literature of rabies in animals supported by laboratory diagnostics dates from 1949 [7], in which the author stated that foxes (both red and arctic) are the main hosts of the virus for the infection of domestic dogs. To prevent the infection of valuable sled dogs, some of these dogs were vaccinated during this outbreak, which lasted from 1945 to 1947, and involved the northern and western coasts of Alaska, as well as Interior Alaska. Former travelling nurse Alma A. Carlson reports on an earlier laboratory diagnosis of rabies in her 1975 recording [20]. She reported on a dog quarantine in Wainwright during her time in Alaska between 1928 and 1942. This quarantine prevented the spread of rabies in the Arctic coast community and involved laboratory diagnosis of rabies in one of the dogs that developed clinical signs of rabies. Laboratory diagnosis of rabies in specimens from these outbreaks was limited to the available methodologies at that time, namely microscopic detection of Negri bodies in neuronal cells of infected animals and mouse inoculation assays. It is now understood that Negri bodies are not readily detectible in all cases of rabies infection and, therefore, under-reporting based on this methodology is likely.

An outbreak involving Interior Alaska (where currently rabies does not occur) was also reported by Rausch in 1958 [18]. That study summarised rabies activity in Alaska from 1949 to 1957 and known cases in the Interior were limited to the years 1949, 1952 and 1953.

## Public health concerns

**Figure 1.**
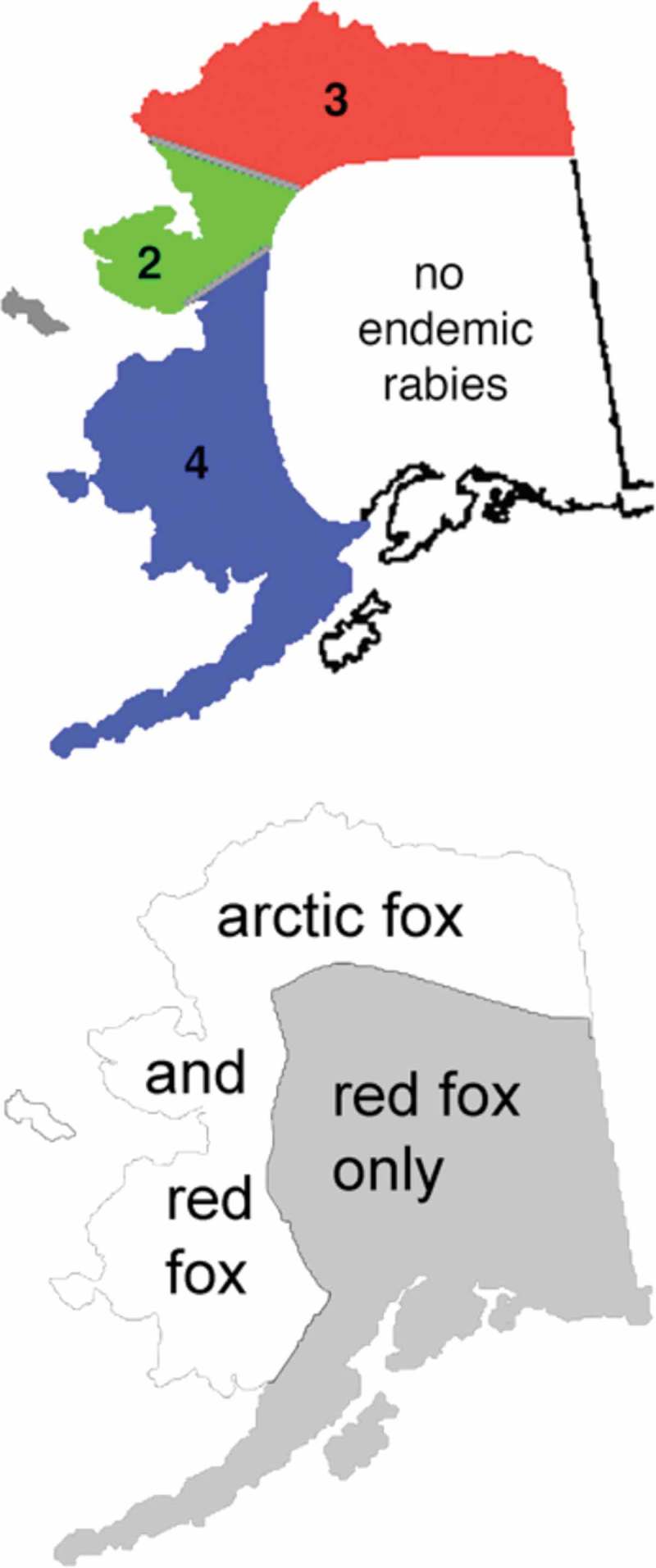
Distribution of rabies (top panel) and foxes in Alaska. Reproduced with permission from Ref. [27]. The numbers refer to the rabies virus variants found in the indicated regions.

**Figure 2.**
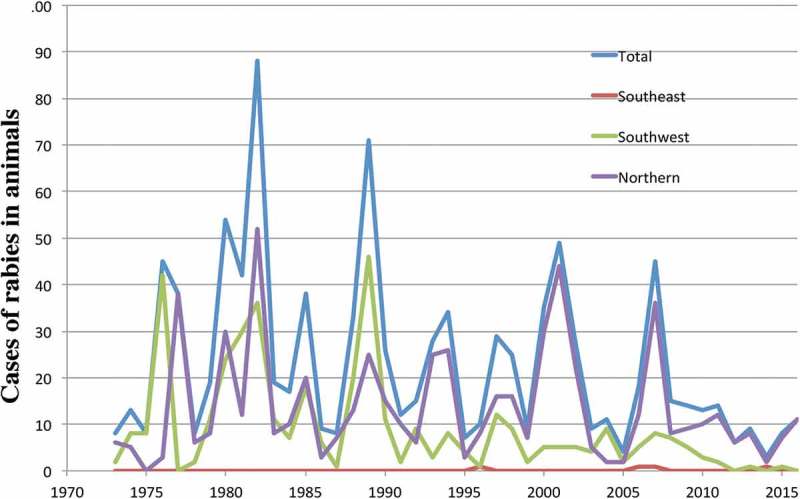
Reported animal rabies cases 1973-2016 by region. Not shown are the Anchorage, Gulf Coast, Interior and Mat-Su regions, which did not provide any cases. Regions are defined as health reporting regions by the State of Alaska (http://www.dhss.alaska.gov/dph/InfoCenter/Pages/ia/brfss/geo_phr.aspx).

In Alaska, rabies still poses a significant public health concern, especially in the face of a changing Arctic [21]. The Northern and Western coastal areas are considered endemic for rabies in both red and arctic foxes (Figure 1). The Section of Epidemiology for the State of Alaska reported 1,004 animals as positive for rabies in their annual disease reports during the period 1973–2016 (http://epibulletins.dhss.alaska.gov). Foxes represent the majority of these cases, but dogs have also tested positive. Over 99% of these animals were from the wildlife rabies-endemic areas of Southwestern and Northern Alaska (Figure 2). No animals were diagnosed with rabies in the areas of high human density including Interior Alaska (Fairbanks) or the greater Anchorage area including the Mat-Su valley. However, the transport of dogs from endemic areas and even foreign countries into urban population centres continues to pose a threat to public health even in non-endemic urban areas [22].

Dog bites are the most frequent events leading to the administration of post-exposure prophylaxis (PEP) in Alaska [23]. The high frequency of domestic dog involvement in the potential exposure of humans to rabies is due to the lack of veterinary services (such as dog population control and vaccination) in remote areas. These rural areas are often also endemic foci for rabies in terrestrial wild mammals (such as foxes), which trappers harvest for their pelts.

The number of dog bites that require hospitalisation of humans is significantly higher in Alaska than the US average [24,25]. In the rabies endemic areas of Northern and Southwest Alaska, the annual bite rate per 100,000 persons was 9.4 and 10.4, respectively, between 1991 and 2002 [25] and 8.5 and 7.0 between 2001 and 2011 [26]. This compares to 3.1 per 100,000 persons estimated dog bites at the national level [26].

In the event of human exposure to the rabies virus, costly measures must be taken immediately to prevent the development of the fatal disease. PEP includes multiple vaccinations and in some cases, the administration of antiglobulin, requiring significant public health resources. From 2002 to 2012, 148 humans received PEP following possible exposure to rabid animals in Alaska [23]. Between 2008 and 2012, all residents receiving PEP were exposed in enzootic areas. Dog bites are the most frequent events leading to the administration of PEP in Alaska [23].

This convergence of the continuous presence of rabies virus in wildlife populations and high rates of severe dog bites highlight the importance of rabies surveillance and prevention in rural areas where significant healthcare disparities exist, and access to both veterinary and human medical services is limited [27].

## Ecology of rabies

To understand the current and future dynamics of rabies in Alaska, one must consider the ecology of hosts and virus in the context of climate and ecosystem diversity in Alaska. The state of Alaska stretches over more than 17 degrees latitude. The southern ecosystem comprises temperate coastal rain forests; in the North, coastal tundra dominates; and the Interior region consists of a continental boreal ecosystem. In addition, the Alaska and Brooks mountain ranges encompass alpine ecosystems, with Denali as the highest mountain in North America. This diversity in ecosystems leads to a variety of unique host–pathogen–environmental interactions, which may differentially influence disease dynamics. These differences are reflected in the variation of predominant host species for wildlife rabies across Alaska. While foxes are the maintenance hosts for rabies in the Northern and Western coastal regions, bats harbour the virus in the southeastern coastal region.

## Rabies in foxes

Enzootic terrestrial rabies is limited to the northern and western part of the state (Figure 1, http://dhss.alaska.gov/dph/Epi/id/SiteAssets/Pages/Rabies/regions.gif). Three different rabies virus strains have been described in this enzootic region. Arctic-2 is found in the area of the Seward Peninsula, while Arctic-3 is restricted to the northern coast. Arctic-4 is only found in the southwestern region of Alaska (Figure 1) [9]. Nothing is known about possible biological differences between these variants such as virulence or adaptations to specific host species. The spatial distribution of variants was found to be relatively stable between 1989 and 2008 [9,28,29]. This stability suggests that the virus is locally maintained between the epizootics that occur every 3–6 years (Figure 2) [30]. Foxes are the main maintenance host, in that they are the species most often reported as rabid, and are likely the only canids that are numerous enough to serve as long-term maintenance hosts in Alaska. Diagnosed cases of rabies in both red and arctic foxes follow similar temporal patterns; years with high numbers of reported cases in red foxes correlate with years in which many cases occur in arctic foxes [30]. The range of the arctic fox mimics the general distribution of endemic terrestrial rabies in Alaska, while the red fox is also present in areas free of endemic rabies. Exceptions to this general correlation are cases of rabies in the western Cook Inlet region and on Alaska Peninsula. However, only very few cases have been reported in these areas that could result from spillover infections into red foxes. Likely, the red fox cannot maintain the Arctic rabies variant in Alaska because of lower population densities compared to the arctic fox or red foxes in more southern areas where this species serves as the main maintenance host of the rabies virus. In addition, the geographic distribution of the arctic fox population more closely parallels the phylogeography of rabies strains, suggesting that this species plays a larger role as a maintenance host than the red fox. However, these conclusions on relative importance of red and arctic foxes in maintenance of rabies should be regarded with some caution as a multi-host system of virus maintenance cannot be excluded [28].

## Rabies in bats

Microbats are another potential vector of rabies virus in Alaska. Seven species of bat have been identified in Alaska (the little brown bat, *Myotis lucifugus*; Keen’s myotis, *Myotis keenii*; the long-legged myotis, *Myotis volans*; the California myotis, *Myotis californicus*; the silver-haired bat, *Lasionycteris noctivagans*; the hoary bat, *Lasiurus cinereus* and the Yuma myotis, *Myotis yumanensis*) with the majority residing in the coastal rainforest ecoregion of the Southeast [31,32]. The little brown bat (*M*. *lucifugus*), the most abundant bat in Alaska, has been identified as far west as the Bering Sea coast, and as far north as 67.41 decimal latitude (north of the Arctic Circle). However, unlike the southeastern species, some of which are believed to be year-round residents, the overwintering behaviour of the little brown bat in more northern latitudes is unknown [33]. Since 1993, five bats have been identified as infected with rabies virus in Alaska: three Keen’s myotis and two little brown bats. Three different rabies strains were typed from these animals, including silver-haired bat (isolated from two little brown bats and a Keen’s myotis), red bat (isolated from a Keen’s myotis) and eastern red bat (isolated from a Keen’s myotis) strains. Notably, all of the rabid bats were collected in the Southeast during the summer and autumn, in relatively populated areas [31]. The true prevalence of bat rabies in the state of Alaska is difficult to determine in that much of the state is uninhabited by people, making human encounters with sick bats infrequent. The silver-haired bat rabies strain is the most commonly reported rabies variant in domestically acquired human rabies cases in the United States[34]. However, no cases of human rabies have been attributed to this variant in Alaska. Nevertheless, the presence of this strain in Alaska bats is concerning. The silver-haired bat rabies strain has been isolated from two myotis species in Alaska (Keen’s myotis and the little brown bat); this may represent a cross-species transmission between an infected silver-haired bat and these two *Myotis* species. Alternatively, it is possible that species misidentification of the myotis bats has occurred; the rabid bats in Alaska have been identified based on morphological criteria, a method which has been reported to misidentify bats approximately 38% of the time, when compared to genetic barcoding [35]. Genetic barcoding (identification of a species by its unique cytochrome *c* oxidase subunit 1 DNA sequence) of the five rabid bats recovered in Alaska will be invaluable to help delineate the pattern of rabies transmission among bats in this state. Although no cases of establishment of bat rabies strains in terrestrial mammal populations have been identified in Alaska, potential for this type of transmission exists, as illustrated by recent outbreaks of bat rabies in skunks (*M*. *mephitis*) and grey foxes (*Urocyon cinereoargenteus*) in Arizona [36]. More extensive research is needed to determine if rabies transmission occurs between bats and other domestic or free-ranging terrestrial vertebrates in Alaska.

## Climate change and host distribution

Shifts of host species ranges in response to climate change lead to changes in population density and spatial distribution of susceptible hosts. A sufficient density of susceptible hosts is necessary for disease maintenance [37], and the spatial structure (including movements) of hosts determines whether a disease can invade other areas in the host’s range [38]. The efficiency of disease transmission is determined by (1) host population density, (2) the degree of host susceptibility, (3) the duration of latent and infectious periods and (4) the rate and distance of host movement. Climate change alters the availability of viable habitat for the hosts. This results in the hosts redistributing their ranges and densities relative to habitat resources. Since multiple host species capable of infection by the same pathogen can have different habitat requirements, a redistribution of host ranges may change the species’ geographic overlap and hence alter the frequency of their interactions. However, the specific effects of these changes on disease ecology are not well understood in the north [39].

## Fox ecology in the context of rabies and climate change

The extensive range of the red fox throughout much of North America has enabled this species to spread rabies from the Arctic interface into southern parts of Canada and the northeastern USA. A major epizootic in the 1950s and 1960s originating in northern Ontario spread as far south as the border to the USA and persisted as the Arctic-1 variant in that province for decades [40], with periodic incursions into the northeastern USA. More recently, waves of infection have spread from the northeastern Canada into parts of southern Canada, even reaching the island of Newfoundland [41]. In Alaska, however, red foxes do not maintain endemic rabies in areas without arctic foxes, even though their range has extensive contact with arctic fox habitat (Figure 1).

Anecdotal and published [42,43] evidence supports a northward expansion by the red fox, which is a specific example of a range expansion of a host species that plays a major role in multiple zoonotic diseases. This expansion has led to interspecific competition with the arctic fox. However, no systematic study on red fox range expansion has been published for Alaska, where red fox predation on arctic foxes has been reported [44,45]. A warming climate and increased human land development, both of which improve red fox habitat conditions, may contribute to the expansion of the northern range limit of the red fox and consequently reduce the arctic fox species range. A change in dominance or the displacement of one carnivore by a different species may have significant epizootiological consequences for rabies, echinococcosis and other zoonotic diseases in the far north. For rabies, the modelling data make it somewhat likely that transmission of rabies virus under the current ecological regime will decrease in a warming climate. This conclusion is also supported by the numbers of reported rabies cases (Figure 2). The numbers show a negative trend between 1973 and 2016 (*p* = 0.03 by ANOVA).

## Bat ecology in the context of rabies and climate change

The roosting behaviour of bats in Alaska, particularly the little brown bat, is different from that noted in the continental United States. Specifically, maternity roosts and overwintering roosts are found mainly in man-made structures, rather than in caves [33]. Presumably, this is due to the additional warmth provided by these structures. However, winter reports of roosting bat colonies are sparse, and the overwintering or migration habits in Alaskan bats are largely unknown. Evidence of year-round bat residence has only been discovered in the warmer Southeast Alaska, where cave use has been noted and involves only small numbers of bats [33]. In that, the rabies virus has only been isolated from bats collected in the Southeast, it is difficult to determine the frequency of rabies virus transmission in colder areas of Alaska, such as the Interior [31]. The low frequency of rabies virus identification in bats in the interior region may be either a function of low population density, sparse or individual roosting behaviour or an actual low rate of infection in these bats. The potential for the rabies virus to overwinter in silver-haired bats has been recently explored, with the finding that the incubation period is prolonged and transmissibility increased in experimentally inoculated bats maintained in simulated hibernation conditions [34]. It is currently thought that the Alaskan interior and north are too cold to encourage overwintering of bats. However, analysis of temperature and precipitation recordings in Alaska has identified a statewide average warming of about 1°C since 1920 [46]. If climate change continues to create warming in the Alaska interior and north, it is possible that more bats may move into these regions and maintain year-round residence through hibernation, thus increasing the potential for rabies transmission in these areas. Although historic range data for bats in Alaska are sparse, the relationship between climate and bat range has been explored in microbat species elsewhere in the Northern hemisphere. For example, the association of climate change with bat range expansion has been recently demonstrated in the Kuhl’s pipistrelle bat (*Pipistrellus kuhlii*). Utilising a model incorporating climate data and Eurasian range maps of this species between 1970 and 2013, it was determined that an increase in mean winter temperature is the most significant factor in the geographic expansion of this species in Eurasia [47]. Similarly, an increase in minimum temperature is predicted to expand the range of Nathusius’ pipistrelle bat (*Pipistrellus nathusii*) in the United Kingdom by twofold over 40 years (2010–2050) according to models incorporating historic climate and bat range data [48].

## Modelling approaches

To assess the ecology of terrestrial rabies in Alaska, two recent studies employed modelling approaches. Kim et al. utilised Poisson regression analysis to identify climatic factors associated with the number of reported rabies cases [30]. This study confirmed the seasonality of reports of rabid foxes with a concentration in the winter and spring, as well as the cyclical nature of reported cases, with increased reports occurring every 3–4 years. The arctic fox rabies reports showed seasonality while number of reported rabies cases in red foxes did not show seasonality. These results suggest that the ecology of rabies in arctic and red foxes differs, and a change in climate could alter the ecology of rabies overall by shifting the disease burden from arctic to red foxes. This possible shift to red foxes as a more important host species rests on the assumption that climate change and human development will negatively affect the arctic fox by providing more resources for the larger red fox, a competitor of the arctic fox. Overall, the authors developed a conceptual model that predicts a reduced risk for human rabies exposure given a scenario of a warming Arctic. Limitations of this study were that it did not investigate rabies dynamics from a spatial perspective. Given the large seize of Alaska, ecological drivers are likely quite different in Northern and Southwestern Alaska. In addition, this study relied on reported cases of rabies only. The detection of rabies is highly biased towards cases close to human infrastructure. This likely sampling bias could explain the higher number of cases diagnosed in red foxes compared to arctic foxes.

Using a different, spatially explicit, modelling approach, Huettmann and colleagues arrived at a similar conclusion with a reduced ecological niche for rabies as measured by confirmed cases, given climate conditions in a warming scenario for the Arctic [49]. Using machine learning approaches, the authors developed a spatial distribution model of relative index of occurrence of rabies in Alaska. The models identified coastal regions in Northern and Western Alaska as well as a region in Interior Alaska as areas that fall into the ecological niche of high likelihood of rabies detection. Environmental parameters most strongly associated with that niche are distance from infrastructure, elevation, distance to coast, precipitation in June, precipitation in February, precipitation in October and temperature in October. When a model based on climate data only was developed, it still followed the general spatial characteristics of the model that included all variables tested. Using this climate-based model and a scenario for climate change, the geographic area of high relative occurrence was reduced especially in southern parts of the state. While this study addressed the spatial limitation of the previously discussed modelling approach, it still largely relied on reported cases of rabies, resulting in the limitations discussed above.

## Challenges

Williams described the challenges of working on infectious diseases in the Arctic in 1949 [7]. Long and cumbersome arctic travel, as well as a sparse human population in this region, made epidemiological studies difficult in the mid-twentieth century. While conditions and technology have improved, the high cost of studies, the relatively low disease impact on large human populations and a lack of infrastructure in remote areas still make studies of rabies in the Arctic challenging. Related to the high cost is the fact that most available information and data are associated with human activity. For example, the nature of rabies surveillance is closely linked to possible exposure of humans. While this focus on areas with higher human activity is rational from an economic and public health standpoint, however, it might lead to significant biases in the data, and it reduces the reliability of modelling approaches based on those data [49]. The inability to reliably predict future human development hinders the estimation of future disease impact in Alaska and the circumpolar region as a whole.

With the potential for dramatic climate and human activity changes in the far North, the effect of these changes on complex disease systems is uncertain and poses new challenges to our understanding of rabies ecology in the Arctic. The complex host–pathogen interactions at high latitudes are not currently well understood, and with a changing climate, disease dynamics are likely undergoing continual change as well, necessitating ongoing study in this region [39].
